# Acute Biliary Septic Shock

**DOI:** 10.1155/1990/71059

**Published:** 1990

**Authors:** Tse-Jia Liu

**Affiliations:** Division of General Surgery Department of Surgery Taichung Veterans General Hospital No. 160, Sec. 3, Chungkang Rd. Taichung 40705 Taiwan

## Abstract

Forty-seven cases of biliary tract infection with septic shock are presented. The sepsis was caused by empyema of the gallbladder in 23 cases and by cholangitis in the remainder. Gallstones were most frequently the cause of the sepsis. An appropriate diagnostic description of the syndrome of biliary tract infection and septic shock should therefore include a description of the underlying biliary disease as well as the term acute biliary shock. In this series, emergency surgical management by removal of gallstones and drainage of suppuration was felt to be the most appropriate treatment. There was a high incidence of gallbladder rupture (10.6%) and intrahepatic stones (53.2%). Of the 13 patients who died, 8 might have survived if early operation had been performed after the diagnosis of acute biliary septic shock was established.


*HPB Surgery*, 1990, Vol. 2, pp. 177–183
Reprints available directly from the publisher
Photocopying permitted by license only

© 1990 Harwood Academic Publishers GmbH
Printed in the United Kingdom

### ACUTE BILIARY SEPTIC SHOCK

TSE-JIA LIU
*Division of General Surgery, Department of Surgery, Taichung Veterans General Hospital*

*(Received 11 September 1989)*

Forty-seven cases of biliary tract infection with septic shock are presented. The sepsis was caused by empyema of the gallbladder in 23 cases and by cholangitis in the remainder. Gallstones were most frequently the cause of the sepsis. An appropriate diagnostic description of the syndrome of biliary tract infection and septic shock should therefore include a description of the underlying biliary disease as well as the term acute biliary shock. In this series, emergency surgical management by removal of gallstones and drainage of suppuration was felt to be the most appropriate treatment. There was a high incidence of gallbladder rupture (10.6%) and intrahepatic stones (53.2%). Of the 13 patients who died, 8 might have survived if early operation had been performed after the diagnosis of acute biliary septic shock was established.

KEY WORDS: Biliary tract infection, septic shock.

Although septic shock is constantly present in 10%–30% of patients with biliary tract infection[1,2], it has never been discussed as a single entity. The commonly used descriptions for these patients, e.g. acute cholangitis or acute suppurative obstructive cholangitis, are not inclusive. Gall bladder empyema, for example, is not covered[3]. Therefore, we use the term "acute biliary septic shock" (ABSS) to designate the special group of patients with this kind of serious biliary tract infectious disease before definite pathological diagnosis obtained. Generally, an apparently higher incidence of morbidity and mortality is observed in ABSS patients than in other patients with biliary tract infections. A retrospective clinical review was carried out to specify this peculiar condition.

### MATERIAL AND METHOD

Patients: From Nov. 1982 to Jul. 1987, a total of 792 patients underwent emergent biliary operations for acute biliary tract infection at Taichung Veterans General Hospital; 47 (5.9%) of them, including 27 males and 20 females, presented with the signs of septic shock and met all of the following criteria, which were proposed by Sprund *et al*[4] with slight modification: (A) systolic pressure less than 90 mmHg or a decrease of 50 mmHg of it in a patient with previous hypertension; (B) evidence of altered mental status or oliguria; (C) irresponsiveness to rapid infusion of 500 c.c. of normal saline; (D) bacteremia of biliary origin which was proved either by positive bile culture for bacteria or frank pus in the biliary system but no other

Correspondence to manuscript and reprints: Tse-Jia Liu, M.D., Division of General Surgery, Department of Surgery, Taichung VGH No. 160, Sec. 3, Chungkang Rd. Taichung, Taiwan, 40705, R.O.C.

infection source had been found. The age of these 47 ABSS cases ranged from 22 to 83 years with an average of 57.6 years. Thirty-one patients had undergone other operations previously, 25 of the operations were related to the gastrointestinal tract (cholecystostomy in 2, cholecystectomy with or without choledochotomy in 13, appendectomy in 2, partial gastrectomy in 7 and abdominoperineal resection of rectal cancer in 1). Twenty-three cases of concomitant diseases were noted, including liver cirrhosis in 8 patients, hypertension in 3, duodenal ulcer in 2, benign prostate hypertension, diabetes mellitus, chronic glomerulonephritis, chronic obstructive pulmonary disease, myasthenia gravis, cardiac arrhythemia, chronic obstructive pulmonary disease, urinary bladder cancer, recurrent breast cancer and gastric cancer in one case respectively. Before the operation, 44 patients with cholelithiasis obtained the exact diagnosis by ultrasound examination. Bile duct obstruction caused by a tumor in 2 cases and fibrotic stricture in 1 patient was confirmed by endoscopic retrograde cholangiopancreaticography (ERCP).

Resuscitation: In addition to routine fluid replacement, all patients received prophylactive broad spectrum antibiotics composing of aminoglycosides, clindamycin or metronidazole and penicillins or cephalosporins preoperatively. Large doses of steriod (Methylprednisolone, Upjohn, 30 mg/kg) were applied in 14 cases during both preoperative and postoperative periods which was randomized in consecutive order for combating shock.

Treatment: An emergent exploratory laparotomy for removal of the septic focus was performed on all patients, 32 of them received the operation within 48 hours after diagnosis of ABSS but the remaining 15 underwent surgery later than 48 hours due to delay in transfers from other hospitals. Thirty-one patients underwent cholecystectomy, choledochotomy and T-tube drainage while another 13 patients received only choledochotomy and T-tube drainage because their gall bladder had been resected. Choledocholithotomy was done in addition if stone was found in the bile duct. Simple drainage of the gall bladder and bile duct was performed on 3 bile duct stricture caused by tumor and fibrosis patients. Eighteen surviving cases with intrahepatic stone received postoperative choledochofibroscopic management for removal of the retained stones.

Statistics: Yate's corrected $\chi^2$ test was used to analyze the difference between the deceased and survived patients.

### RESULT

Symptoms and signs: As shown in Table 1, all of the ABSS patients had abdominal pain and half of them had nausea and vomiting. Although all 47 cases had biliary infection, only 5 cases developed hypothermia and 3 normal body temperature. Charcot's triad[5] was observed in 40 patients and Reynolds' pentad[6] in 25 cases. Three cases presented with a soft, flattened and nontender abdomen on physical examination, whereas the others had varied degrees of peritoneal irritation. Five patients had gall bladder perforation which was impossible to be correctly diagnosed preoperatively; all showed signs of localized or generalized peritonitis on preoperative physical examination.

Laboratory findings: Table 2 shows 16 cases had elevated LDH (240 u/L), 11 of them, including 6 out of 8 cases of liver cirrhosis, expired eventually. Therefore, liver cirrhosis and elevation of serum LDH values had statistical significance of the

**Table 1** Symptoms & Signs

| Symptoms | No. | Signs | No. |
|---|---|---|---|
| Abdominal pain | 47 | Hyperthermia (>38°C) | 40 |
| Nausea and vomiting | 25 | Hypothermia (<36°C) | 5 |
| General weakness | 24 | Jaundice | 38 |
| Backache | 4 | Mentality change | 24 |
| Clay-colored stool | 4 | Charcot's triad | 40 |
| Pruritus | 4 | Reynolds' pentad | 25 |

**Table 2** Laboratory data

| | | | |
|---|---|---|---|
| WBC>10,0000 | 40 (12) | Serum bilirubin>2 mg% | 33 (12) |
| HgB<10 gm% | 12 (4) | SGOT>20 U/L | 29 (9) |
| LDH>240 U/L | 16 (11) | SGPT>20 U/L | 31 (10) |
| Anterial blood pH<7.35 | 22 (6) | Serum amylase >200 U/L | 5 (1) |
| Alk-ptase>200 U/L | 20 (7) | Creatinine>1.5 mg% | 17 (6) |

( ): no. of mortality risk of mortality (Yate's corrected $\chi^2$ test, $p<0.05$ and $<0.001$ respectively). The other factors, e.g. leucocytosis, hyperbilirubinemia, anemia, thrombocytopenia, metabolic acidosis, hyperamylasemia, azotemia, elevation of liver transaminase enzymes and alkaline phosphatase, were not related to the treatment failure.

Steroid administration: There were 4 mortalities in 14 patients to whom large doses of steroid were administered and 9 deaths in 33 cases who had no steroid therapy. No statistical difference could be found between these two groups ($p>0.5$).

Operative findings: As shown in Table 3, 35 out of 44 cases of cholelithiasis were found to have suppuration in the bile duct during operation. One patient with a metastatic breast tumor compressing the lower common bile duct also had purulent fluid in the bile duct. There were 23 cases of gall bladder empyema, and 17 of them had concomitant suppuration in the bile duct. Five out of these 17 patients had gall bladder perforation simultaneously; 4 expired after treatment. Among 25 patients with intrahepatic duct stones, 4 had an entirely normal appearance of extrahepatic biliary tree; however, 3 such cases still had suppuration behind the stones in the liver. Five cases had gall bladder stone only, and 4 had gall bladder empyema. There was neither bile duct obstruction nor cholangitis, although the septic focus was in the biliary tract. Three of these 5 patients who received an immediate operation survived but another two who underwent a delayed (70 hours) surgery died eventually.

Bacteriology: Fifteen cases referred from other hospitals had been treated by antibiotic therapy so no bacterial growth in the blood culture was found. Table 4 illustrates E. coli was the most common aerobic pathogen in the bile and blood culture; other frequently found pathogenic bacteria were Klebsiella and Pseudomonas. No obvious correlation was noted among the mortality rate, the number of isolated bacteria strain and species of bacteria. Four incidences of anaerobic bacteria, including bacteroides fragilis in 3 and peptostreptococci in 1, were isolated from the bile culture while no anaerobic bacterium was obtained from

**Table 3** Operative Findings

| Pathology | No. | Suppuration in GB & BD | | GBP |
|---|---|---|---|---|
| GBS | 5 (2) | 4 (2) | — | — |
| GBS + CBDS | 7 (1) | 7 (1) | 5 (1) | 2 (1) |
| GBS + CBDS + IHDS | 6 (2) | 5 (1) | 6 (2) | 1 (1) |
| CBDS | 7 (3) | 2 | 7 (3) | — |
| GBS + IHDS | 3 | 2 | 2 | — |
| CBDS + IHDS | 11 (3) | 2 (2) | 11 (3) | 2 (2) |
| IHDS | 5 (1) | — | 3 | — |
| CBDS Stricture | 1 | — | — | — |
| Tumor, low CBD | 2 (1) | 1 (1) | 1 (1) | — |
| Total | 47 (13) | 23 (7) | 35 (10) | 5 (4) |

S: stone

GB: gall bladder

BD: bile duct

( ): no. of mortality

CBD: common bile duct

IHD: intrahepatic duct

GBP: gall bladder perforation

**Table 4** Bacteriology

| Result | Bile | Blood |
|---|---|---|
| *E. Coli* | 32 (9) | 13 (4) |
| Klebsiella | 16 (5) | 7 (3) |
| Pseudomonas | 13 (5) | 6 (3) |
| Citrobacter | 8 (3) | 3 (1) |
| Enterococcus | 6 (2) | 4 (1) |
| Proteus | 6 (1) | 0 (0) |
| Enterobacter | 1 (1) | 1 (0) |
| Serratia | 1 (1) | 0 (0) |
| Strept. nonhemolyticus | 1 (0) | 0 (0) |
| Candida | 1 (1) | 1 (1) |
| Bacteroides fragilis | 3 (1) | 0 (0) |
| Peptostreptococcus | 1 (0) | 0 (0) |
| No growth | 5 (1) | 21 (4) |
| Single strain | 15 (2) | 6 (3) |
| Mixed strain | 27 (10) | 20 (6) |

( ): no. of mortality the blood culture. Anaerobic infection (bacteroides) mixing with E. coli in the bile culture was found in 1 expired case.

Morbidity and mortality: As shown in Table 5, the most common operative complication was wound infection. Thirteen cases (27.6%) expired after operation, 5 (15.63%) mortalities occurred in 32 cases who received the operation within 48 hours after diagnosis was made; 2 were in the terminal stage of liver cirrhosis and 2 were in moribound condition caused by profound septic shock on their arrival and one died of a sudden attack of cardiac arrhythemia. Eight (53.33%) out of 15 cases undergoing a delayed operation expired; they all died of irreversible septic shock with multiple organ failure. Statistically, early operation provided less mortality rate ($p<0.05$). A detailed analysis of the relation between mortality and the location of frank pus (Table 6 ) disclosed that expired cases were positively

**Table 5** Postoperative Complications

| | | | |
|---|---|---|---|
| Wound infection | 6 (1) | Duodenal leak | 1 (1) |
| Minimal bile leak | 3 (0) | Stress ulcer bleeding | 1 (1) |
| Wound disruption | 2 (2) | Pneumonia | 1 (1) |
| Hepatic failure | 2 (2) | Acute renal failure | 1 (0) |

( ) number of mortality

**Table 6** Location of Frank Pus

| gall bladder | bile duct | Total | Mortality |
|---|---|---|---|
| + | + | 17 | 5 |
| − | + | 18 | 5 |
| + | − | 6 | 2 |
| − | − | 6 | 1 |

correlated with pus in the bile duct but not in the gall bladder. Nevertheless, among the 6 cases who did not have obvious suppuration in the whole biliary tract, 1 mortalities still ensued.

### DISCUSSION

According to the disease severity of cholangitis, 3 designations are usually employed: acute cholangitis, acute suppurative cholangitis and acute suppurative obstructive cholangitis, with the last one denoting the cholangitis cases with septic shock[1]. In a study of acute cholangitis, Boey and Way disclosed that the shock symptom was neither a necessity in cases of suppurative cholangitis nor invisible in non-suppurative ones[7]. Their conclusion was verified in our ABSS cases, in which 6 patients who did not have frank pus in the biliary tract still presented with typical symptoms of septic shock and 1 of them died ultimately. In addition, Reynolds' pentad, which was pathognomonic for suppurative cholangitis with septic shock, was observed only in 50% of the ABSS patients. Our study as well as other reports[2,3] showed that simple gall bladder empyema, either with or without gall bladder stone, e.g. acute acalculous cholecystitis[8], could cause typical septic shock and even mortality. Benign stricture and malignant obstruction of bile duct with superimposed infection may also result in ABSS[1,7]. From the clinical and pathological view-points, ABSS is more suitable than any other terms for describing a biliary infection involving septic shock. A reasonable and proper description for a biliary infection with septic shock before obtaining the definite pathological diagnosis at biliary tract in fact should include the underlying biliary disease and status of infection; e.g. gall bladder stone with ABSS, CBD stone with ABSS, obstructive jaundice with ABSS, etc.

The clinical manifestations and laboratory examinations of ABSS patients illustrated the typical features of acute biliary infection. Abdominal ultrasound and CT scan have been widely used for detecting gall stone disease; however, detailed history taking and thorough physical examinations are very useful for a patient with ABSS. Gall bladder perforation can be suspected preoperatively only by the presence of peritonitis and be confirmed by exploratory laparotomy but hardly by imaging procedures. Resuscitation by the use of steroid drugs for septic shock is very controversial in general[4,9]. In this study it proved statistically to have no effect on treating ABSS.

Cholelithiasis and bile duct stricture are the commonest underlying pathogenesis in biliary tract infections. When the infections progress into septic shock, gall stone is the infectious focus in the majority of cases[1,7]. In the present series, all ABSS patients except 3 had gall stones in the biliary tree. Thirty-two percent of the studied patients had previous surgery for cholelithiasis and they all developed ABSS due to recurrence of their original disease. We also frequently found purulent discharge behind the stones in the liver. To eliminate or minimize the continuous infection, adequate drainage of suppuration and complete removal of gall stones whether inside or outside the liver constitute the basic therapeutic modality for treating ABSS patients. A thorough exploration of the whole biliary system to eradicate the hidden stones in the liver with suppuration behind is advisable for oriental cases of cholelithiasis with ABSS because one third to half of our patients were found to have liver stone[10]. Currently, either percutaneous transhepatic cholangiography and drainage or ERCP with drainage is a very useful therapeutic modality for decompression and drainage of purulent fluid in the bile duct[11,12], but neither is good for removal of IHD stones nor treating the perforated gall bladder.

In treating the biliary infection with resulting septic shock, the death rate of 30% was common ensued by either surgical[1,7] or non-surgical interventions[11,12]. A significant difference in the mortality rate was observed in the two groups of patients who received an early or a delayed operation (5/32 vs 8/15, $p<0.05$). All the expired patients who received a delayed operation died of irreversible septic shock and multiple organ failure, while all the deceased cases who underwent an operation within 48 hours were either moribound on admission or died of unrelated conditions. Welch's suggestion that an emergent operation is absolutely necessary for treating acute biliary septic shock patients was confirmed in our study[13]. Hence, it is very obvious that early detection and early operation might have saved some expired ABSS patients and reduced the high mortality incidence in this series.

### *References*

1. Pitt HA, Longmire Jr. WP (1980) Suppurative cholangitis. In: Hardy, JD. (Editor) Critical Surgical Illness. 2nd edition. Philadelphia. W.B. Saunders Co. pp: 380–408.
2. Faber RG, Ibrahim SZ, Thomas DM, Beynon GPJ, Le Quesne LP (1987) Gallstone disease presenting as septicaemic shock. *Br J Surg* **65**, 101–105.
3. Thornton JR, Heaton KW, Espiner HJ, Eltringham WK (1983) Empyema of the gall bladder - reappraisal of a neglected disease. *Gut* **24**, 1183–1185.
4. Sprung CL, Caralis PV, Marcial EH, et al (1984) The effect of high-dose corticosteroids in patients with septic shock; A prospective, controlled study. *N Eng J Med* **311**, 1137–1143.
5. Charcot JM, Oeuvres Compleetes de JM Charcot (1891) VI. Lecons sur les maladies due foie et des reins. Bourneville, Sevestre et Brissau, Paris: Vve Babe et Cie. pp: 194.
6. Reynolds BM, Dargan EL (1959) Acute obstructive cholangitis, a distinct clinical syndrome. *Ann Surg* **150**, 299.
7. Boey JH, Way LW (1980) Acute cholangitis. *Ann Surg* **191**, 264–270.
8. Glenn F, Becker CG (1982) Acute acalculous caholecystitis: an increasing entity. *Ann Surg* **195**, 131–6.
9. Connors RH, Coran AG, Drongowski RA, Wesley JR (1983) Combined fluid and corticosteroid therapy in septic shock in puppies. *World J Surg* **7**, 661–668.

10. Choi TK, Wong J, Ong GB (1982) The surgical management of primary intrahepatic stones. *Br J Surg* **69**, 86–90.
11. Lygidakis NJ (1982) Acute suppurative cholangitis: comparison of internal and external biliary drainage. *Am J Surg* **143**, 304–306.
12. Cotton PB (1985) Non-surgical treatment of biliary disorders: endoscopic approach. In: Berk JE (Ed.) Bockus Gastroenterology Vol. 6, 4th Ed. Philadephia. W.B. Saunders Co. pp: 3732–3741.
13. Welch JP, Donaldson GA (1976) The urgency of diagnosis and surgical treatment of acute supprative cholangitis. *Am J Surg* **131**, 527–532.

*Accepted by Miles Little on 11 September 1989.*

### INVITED COMMENTARY

The present paper supports the value of early therapeutic intervention in patients with biliary infection. It has previously been shown that early cholecystectomy in patients with acute cholecystitis results in a reduction of hospital stay and costs without an increase in intra- and postoperative complications as compared to patients initially treated conservatively followed by delayed elective surgery [1,2]. About 10% of patients in the present material, admitted with signs of septic shock proved to have a ruptured gallbladder. Bile peritonitis seen in association with acute cholecystitis is still associated with a 10–20% mortality [3-5] and there is a significant correlation between the time of surgery and death, i.e. the longer the hospital delay before surgery the higher mortality [3]. Mortality in this group of patients was also mainly due to multiple organ failure in the late postoperative period, as was seen in the present study. The need for establishing a new syndrome of biliary tract infection and septic shock, "acute biliary septic shock" seems, however, somewhat unnecessary. Septic shock is a wellknown complication and often the presenting sign of a severe biliary infection, as well as abscess/sepsis of other intraabdominal origin. The key towards increased survival and a lowered morbidity seems to be early and appropriate intervention with the aid of all those diagnostic measures that now are available.

Roland Andersson & Stig Bengmark
Department of Surgery
Lund University
S-221 85 LUND
Sweden

### *References*

1. Van der Linden, W. and Edlund, G. (1981). Early versus delayed cholecystectomy: the effect of a change in management. *Br. J. Surg.*, **68**, 753.
2. Norrby, S., Herlin, P., Holmin, T., Sjödahl, R. and Tagesson, C. (1983). Early or delayed cholecystectomy in acute cholecystitis; A clinical trial. *Br. J. Surg.*, **70**, 163.
3. Andersson, R., Tranberg, K-G., Bengmark, S. Bile peritonitis and acute cholecystitis. *HPB Surgery,* in press.
4. Felice, P.R., Trowbridge, P.E. and Ferrara, J.J. (1985). Evolving changes in the pathogenesis and treatment of the perforated gallbladder. *Am. J. Surg.*, **49**, 466.
5. Larmi, T.K.I., Kairalouma, J.J., Junila, J., Laitinen, S., Ståhlberg, M. and Fock, H.G. (1984). Perforation of the gallbladder. *Acta. Chir. Scand.* **150**, 557.

